# Balancing Feed-Forward Excitation and Inhibition via Hebbian Inhibitory Synaptic Plasticity

**DOI:** 10.1371/journal.pcbi.1002334

**Published:** 2012-01-26

**Authors:** Yotam Luz, Maoz Shamir

**Affiliations:** 1 Department of Physiology and Neurobiology, Ben-Gurion University of the Negev, Beer-Sheva, Israel; 2 Department of Physics, Ben-Gurion University of the Negev, Beer-Sheva, Israel; Gatsby Computational Neuroscience Unit, University College London, United Kingdom

## Abstract

It has been suggested that excitatory and inhibitory inputs to cortical cells are balanced, and that this balance is important for the highly irregular firing observed in the cortex. There are two hypotheses as to the origin of this balance. One assumes that it results from a stable solution of the recurrent neuronal dynamics. This model can account for a balance of *steady state* excitation and inhibition without fine tuning of parameters, but not for *transient* inputs. The second hypothesis suggests that the feed forward excitatory and inhibitory inputs to a postsynaptic cell are already balanced. This latter hypothesis thus does account for the balance of transient inputs. However, it remains unclear what mechanism underlies the fine tuning required for balancing feed forward excitatory and inhibitory inputs. Here we investigated whether **inhibitory synaptic plasticity is responsible for the balance of transient feed forward excitation and inhibition**. We address this issue in the framework of a model characterizing the stochastic dynamics of temporally anti-symmetric Hebbian spike timing dependent plasticity of feed forward excitatory and inhibitory synaptic inputs to a single post-synaptic cell. Our analysis shows that inhibitory Hebbian plasticity generates ‘negative feedback’ that balances excitation and inhibition, which contrasts with the ‘positive feedback’ of excitatory Hebbian synaptic plasticity. As a result, this balance may increase the sensitivity of the learning dynamics to the correlation structure of the excitatory inputs.

## Author Summary

One of the longstanding enigmas in neuroscience is the origin of inherent neural noise. It has been suggested that this noise results from a careful balance of excitatory and inhibitory inputs to neurons. Obtaining this balance requires fine tuning of the relative strengths of the inhibitory and excitatory inputs to each cell. However the mechanism that enables this fine tuning of parameters remains unclear. We suggest that a balance of excitatory and inhibitory inputs can be achieved via a process of unsupervised learning of the inhibitory synaptic strengths. We find that whereas Hebbian learning induces strong positive feedback on excitatory inputs that acts as a force that pulls them towards their boundaries, Hebbian learning of inhibition induces negative feedback. This negative feedback acts to balance the average excitatory input to the cell and makes the learning process less sensitive to the statistics of the inhibitory inputs. Surprisingly, this balance increases the sensitivity of learning to the statistics of the excitatory inputs. Thus, the balance of feed-forward excitation and inhibition emerges as a natural outcome of Hebbian learning applied to the inhibitory inputs to the cell.

## Introduction

### Balance of feed forward excitation and inhibition in the cortex

There is a striking difference between the number of synaptic contacts received by a typical cortical cell, which is about 3,000 to 10,000, and the required number of about 30 excitatory inputs to bring the cell to its firing threshold [1], [2]. For example, inputs from 1,000 excitatory presynaptic cells firing at a medium rate of 10 spikes/s will yield 100
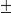
10 (mean 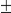 standard deviation) input spikes every 10 ms. At this high level of input, the postsynaptic cell is expected to saturate its firing rate. In addition, whereas *in vitro* experiments have shown that cortical cells fire relatively regularly, *in vivo* recordings reveal a highly irregular neural response [1]. However, fluctuations in the excitatory postsynaptic current are expected to be negligible relative to their mean, and therefore they themselves cannot account for the irregular firing of the postsynaptic cell, as observed *in vivo*.

The prevalent explanation for these seemingly contradictory findings is that excitatory and inhibitory inputs to cortical cells are balanced; i.e., the mean excitatory and inhibitory inputs to the cell are on the same order of magnitude. In terms of the balance hypothesis, the firing of the postsynaptic cell in the above example is not determined by the mean input of 100 excitatory postsynaptic potentials every 10 ms, which will be canceled by the mean inhibitory input, but rather by the fluctuations that are an order of magnitude smaller. Thus, the firing rate of the postsynaptic cell will not saturate and will be characterized by a high degree of variability.

There is, however, some confusion as to the nature and origin of the balanced state. One approach to this problem suggested that this balance results from recurrent neural dynamics [3]–[7]. Tsodyks and Sejnowski [3] showed that feedback (i.e., recurrent interactions) in finite chaotic networks can produce the desired balance. However, this solution requires strong interactions between the neurons and assumes a high probability of synaptic failure. Van Vreeswijk and Sompolinsky [6], [7] showed analytically that the balance of *steady state* excitation and inhibition can be obtained as a stable solution to the network dynamics, and requires no special fine tuning of parameters due to the feedback from the network dynamics. These studies focused on the balancing of the lateral inputs via feedback of recurrent interactions and ignored the feed forward inputs to the system. However, empirical findings have shown there is a fast balance of *transient* inputs to a barrel cortical cell [8], [9]. An alternative model suggested by Newsome and Shadlen [2], argues for the importance of balancing *feed forward* excitation and inhibition inputs in feed forward (propagating) neural networks. However, it remains unclear what mechanism underlies the fine tuning required for balancing feed forward excitatory and inhibitory inputs.

### Spike-timing-dependent plasticity of excitatory synapses

#### Experimental characterization

**Figure 1:**
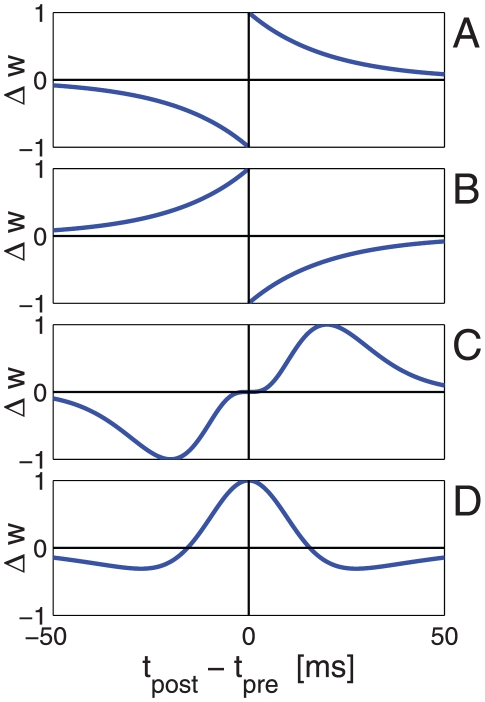
Illustration of different types of STDP curves. The synaptic weight modification as a function of a  caricature of four cases. **A** Hebbian temporally asymmetric STDP, e.g., Bi and Poo [11] for an excitatory synapse. **B** Anti-Hebbian temporally asymmetric STDP, e.g., [21], [22]. **C** Hebbian temporally asymmetric STDP - inhibitory (entorhynal) cortical synapse, e.g., Haas et al. [50]. **D** Hebbian temporally symmetric STDP - inhibitory hippocampal synapse, e.g., Woodin et al. [49].

The overwhelming majority of scientific works on synaptic plasticity has focused on excitatory synapses. It is generally believed that synaptic plasticity is the basis for learning and memory. According to Hebb's rule [10], which remains the foundation of current assumptions on the nature of learning and memory, the interaction strength between two cells that are co-activated will facilitate synaptic efficacy. This rule has been extended to the temporal domain, where it is known as spike-timing-dependent plasticity (STDP). In many cases, the following causal relationship is assumed to exist: an excitatory synapse undergoes long-term potentiation if presynaptic firing precedes postsynaptic firing, and long-term depression is induced when the temporal order of firing is reversed [11]–[20]; this relationship is termed ‘temporally asymmetric Hebbian spike timing dependent plasticity’, Figure 1A (but see also [21], [22] for examples of temporally asymmetric *anti* Hebbian plasticity, Figure 1B).

#### Theoretical characterization

Considerable theoretical effort has been devoted to studying the characteristics of STDP learning of excitatory synapses [23]–[43]. The canonical STDP rule shown in Figure 1A induces positive feedback in the following sense. If a certain synaptic weight is large, a presynaptic spike is more likely to elicit firing of the postsynaptic cell, following firing of the presynaptic cell. This will strengthen the synaptic weight, according to the STDP rule, which, in turn, will increase the likelihood of eliciting firing of the postsynaptic cell following firing of the presynaptic cell. On the other hand, if the synaptic weight is weak, then pre- and post-firing will be uncorrelated and the learning process will sample the STDP curve randomly. If the total depression (area under the acausal branch: post firing before pre) is larger than the total potentiation (area under the causal branch), then the synapse will be further weakened. Theoretical studies have shown that this positive feedback generates a bimodal distribution of excitatory synaptic weights. Note that this bimodal distribution of synaptic weights will exist for a limited range of parameters. Other choices of parameters will cause all the weights to cluster around either their upper or lower boundary. Further investigation has shown that a unimodal distribution can be obtained by scaling the amount of plasticity with the synaptic weight [40], [41], [42], [43].

Theoretical studies have also explored the development of neuronal response properties [28], [29], [36], [39], [42]. In the absence of a reward signal, the STDP rule acts as an unsupervised learning algorithm. In unsupervised learning, the postsynaptic cell ‘learns’ salient features of the statistics of the presynaptic cell's activity, such as the correlation structure. Correlated synaptic inputs from a large group of cells are more likely to cause the postsynaptic cell to fire and, hence, strengthen their synaptic weights, whereas the STDP rule induces competition between different groups of correlated presynaptic cells. The origin of correlated activity of presynaptic cells in primary sensory regions reflects shared preferences for external stimuli. These results have been used to explain the development of ocular dominance columns. Recent theoretical studies have also managed to investigated learning of *recurrent* excitation [44]–[47].

### Spike-timing-dependent plasticity of inhibitory synapses

In contrast to the considerable number of empirical studies on excitatory synaptic plasticity, much less is known about inhibitory plasticity. Nevertheless, evidence for STDP of inhibitory synapses is beginning to emerge [48]–[56]. Woodin et al. [49] found that in hippocampal cultures and acute hippocampal slices, inhibitory synapses are potentiated if pre- and post-spikes are paired to fire within about 20 ms of each other and are depressed when the time difference is larger than about 20 ms, irrespective of the order of firing (e.g., similar to the STDP curve illustration in Figure 1D). In the entorhinal cortex, Haas et al. [50] reported a temporally asymmetric Hebbian STDP rule, in which an inhibitory synapse is potentiated if the inhibitory presynaptic cell fires 5–15 ms before the postsynaptic cell and is depressed if the postsynaptic cell fires 5–15 ms before the presynaptic cell, Figure 1C.

Here we investigate the hypothesis that the temporally asymmetric Hebbian STDP of inhibitory synapses is responsible for the balance of transient feed forward excitation and inhibition. This issue is addressed in the framework of a stochastic dynamical model for learning feed forward inputs to a single postsynaptic cell, and then illustrated using numerical simulations or the learning dynamics of feed forward connections onto an integrate and fire neuron. We start by analyzing the stochastic learning dynamics of a single inhibitory synapse. Then we turn to investigate the learning dynamics of a population of inhibitory feed forward synapses. Finally we study a model that incorporates learning of both feed forward excitatory and inhibitory inputs to a single postsynaptic cell.

## Results

### Learning of a single inhibitory synapse

Considerable theoretical attention has been devoted to the study of learning a single excitatory synapse, see e.g., [41], [43], and in particular the distribution of resultant synaptic weights. Two forces work to shape this distribution. The first is the positive feedback of learning an excitatory synapse (see above) that pushes the synaptic weights to their high and low saturation boundaries and hence contributes to a bimodal distribution. The second is the weight dependence of the STDP rule, which is able to decrease the strength of the positive feedback close to the saturation boundaries and hence contributes to a unimodal synaptic distribution. Following the results of Haas et al. [50] (but see also Woodin et al. [49]), we studied a family of temporally asymmetric STDP rules for the inhibitory synaptic weight 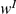 of the form:
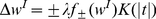
(1)where 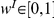 is the dynamic variable that describes the synaptic strength, 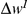 is the change in the synaptic strength following pre (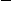) or post (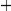) synaptic firing, 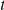 is the time difference between the pre- and post-synaptic firing, 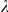 is the learning rate, and 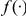 and 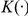 are the weight dependence and temporal filter of the STDP rule, respectively. For convenience we adopted a notation similar to that of [42]. Equation (1) defines the synaptic change due to a single pair of pre-post spikes. We shall assume that the STDP rule is additive with respect to all pairs of pre-post spike times. Equation (1) describes a temporally asymmetric STDP rule, as reported in Haas et al. [50]. The temporal filter, 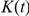, can be modeled by a decaying exponent with a characteristic timescale of about 20 ms (see Figure 1A, e.g., [11]), or by a gamma distribution, similar to the results of Haas et al. [50] (see Figure 1C). For concreteness throughout the paper in all of our numerical simulations we used 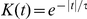 with 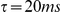. Note that the STDP rule is temporally asymmetric and not antisymmetric due to the different scalings of depression and potentiation in 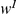, which is expressed in the 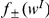 dependence of the synaptic update rule. The structure of 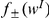 is somewhat less clear from the empirical literature; thus, for convenience of analysis, we adopted the formulation in [42], which generalizes, e.g., [23], [41] (but see [27]):
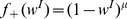
(2)

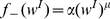
(3)where 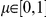 is a parameter that characterizes the weight dependence of the STDP rule. Following equation (1), changes in the synaptic weight, 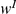, occur only at times where either pre or post synaptic cells have fired:
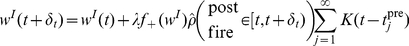
(4)

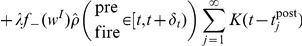
where 
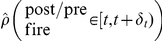
 is a stochastic variable which is one if the post/pre fired at time interval 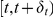 and zero otherwise; 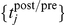 are the spike times of the post/pre synaptic neuron, respectively; the summation is over past times: 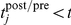. Note that the summation over all past spike times results from our assumption that the synaptic update rule, equation (1), is additive with respect to all pre-post spike time pairs. Taking the short time limit, 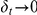, yields
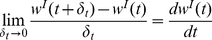
(5)

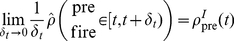
(6)where 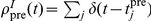 describes the spike train of the inhibitory pre-synaptic neuron in terms of a series of delta function pulses at the spike times of the cell, 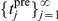 (the summation is over all the spike times). Similarly 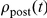 describes the post synaptic spike train. We obtain:
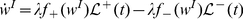
(7)

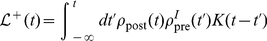
(8)

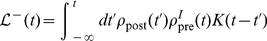
(9)


In the limit of slow learning rate, the synaptic weight, 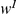, is relatively fixed over long periods of time, 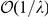, during which the right hand side of equation (7) is sampled by the dynamics such that we can neglect its fluctuations around its mean in the limit of 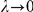. This approximation yields deterministic dynamic equations for the mean synaptic weights:
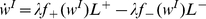
(10)

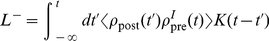
(11)

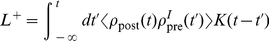
(12)where 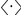 denotes averaging with respect to the distribution of the neural firing, for a given fixed synaptic weight, 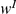. To proceed with the analysis we need to specify the cross-correlation function between the pre-synaptic input and the post-synaptic response, and in particular its dependence on the synaptic weight, 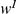. However, the calculation of dependence of the temporal structure of the pre-post firing probability on the synaptic weight, even in the simple case of an integrate and fire neuron is a first-passage time problem [57], [58], which is not a trivial task. Recently, Ostojic et al [59] succeeded in analyzing the cross-correlation function between two integrate and fire neurons assuming the synaptic coupling is sufficiently weak such that the firing rate of the postsynaptic cell can be approximated by a linear function of the presynaptic input. Similarly, in this section, we assume that the synaptic coupling is sufficiently weak such that we can approximate the postsynaptic firing rate by a linear function of the presynaptic input (see also [43], [60], [61]), yielding
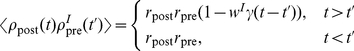
(13)where 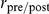 is the pre/post synaptic mean firing rate; and the function 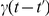 describes the decrease in the conditional firing rate of the postsynaptic neuron at time 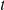 following an inhibitory input spike at time 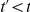.

Substituting equation (13) into equation (10) one obtains:
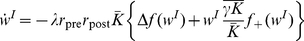
(14)where 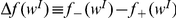, and 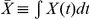. This model highlights the following three major differences between the dynamics of inhibitory and excitatory synapses.

1. The temporally asymmetric Hebbian STDP rule, equation (1), yields a *negative feedback*, which is characterized by a unimodal distribution for the inhibitory synapses. This contrasts with the temporally asymmetric Hebbian STDP rule for an excitatory synapse, which yields a positive feedback and allows for bi-stable solutions.
2. As there is only one stable fixed point for the drift velocity, which is stable for all 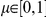, there is no theoretical need for 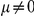, which was introduced to weaken the positive feedback of the excitatory STDP. Hence, we can take 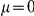.
3. The relative strength of the depression needs to be weaker than the potentiation; i.e., in the 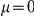 case, the ratio 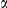 of the area under the acausal and the causal branches of the STDP curve needs to be 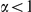, to prevent decay of all inhibitory synapses to zero.

#### Numerical simulations of inhibitory STDP

**Figure 2:**
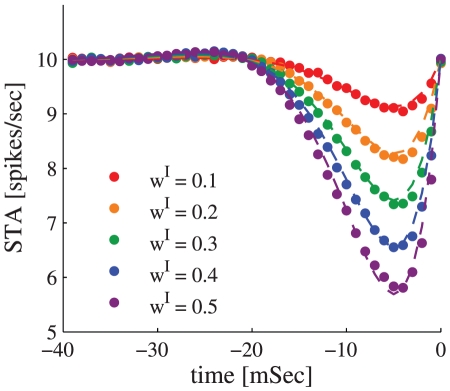
Spike triggered average of inhibitory presynaptic cell. The conditional mean firing rate of the inhibitory presynaptic cell given the postsynaptic cell has fired at time 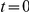, is plotted as function of time, for different values for the strength of the presynaptic weight 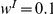, 0.2, 0.3, 0.4, and 0.5 in red, orange, green, blue and purple circles, respectively. The dashed lines show the fits of the form 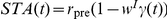 with 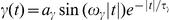. The parameter 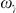 was set to match the zero crossing point of 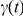, and we optimized the fit over the parameters 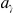 and 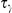.

To test our results beyond the analysis of the above simplified model we performed numerical simulations of the learning dynamics of a feed forward inhibitory synapse to a conductance based integrate and fire postsynaptic neuron (see Methods for details). Figure 2 shows the spike triggered average firing rate of a single presynaptic inhibitory cell as a function of time relative to the firing of the postsynaptic cell (negative times imply pre fired before post), for different fixed values (i.e., without learning) of the synaptic coefficient strength 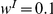, 0.2, 0.3, 0.4, and 0.5 in red, orange, green, blue, and purple circles, respectively. The dashed lines show fits of the form 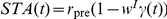 with 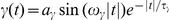. For short times preceding the postsynaptic firing rate the conditional mean firing rate of the inhibitory presynaptic neuron is less than its marginal mean (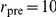 spike/s in the specific example of Figure 2). This decrease is approximately linear in the synaptic weight; whereas for long times, the spike triggered average converges to 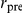. Hence, equation (13) provides a fair description of the pre-post correlations.

**Figure 3:**
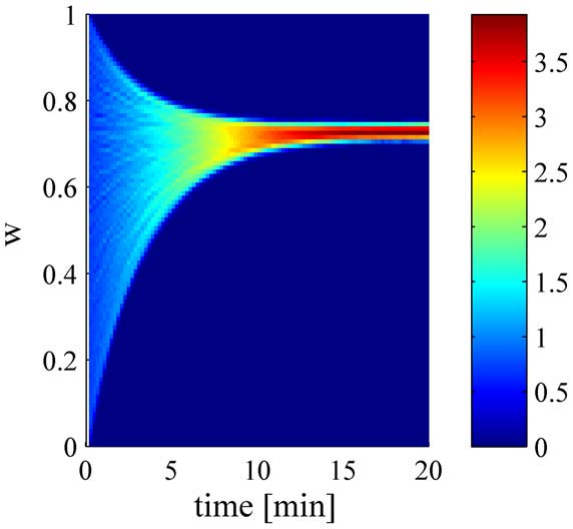
The dynamics of the synaptic weight distribution. The probability *density* of the synaptic weight, 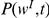 is shown in color code as a function of time. The range of values of 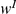, 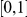, was divided into one hundred equally sized bins, and the probability of having a value in a corresponding bin of size of 1/100 was estimated numerically. The color scale is shown in terms of 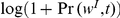. The stochastic learning dynamics of a single inhibitory synapse was simulated using an integrate and fire model (see Methods). The probability density was estimated from the simulations by averaging over 1999 repeats with different realizations for the noise (stochasticity of the presynaptic neurons' firing) and with initial conditions that were uniformly spaced in the interval (0, 1). Here we used 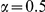, 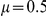, and 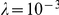.

**Figure 4:**
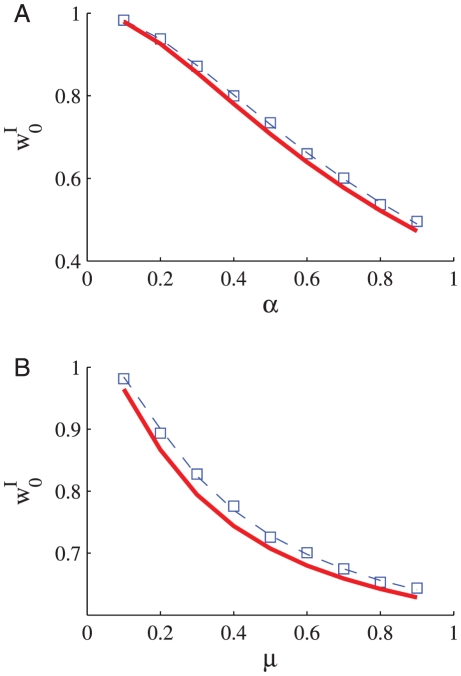
The asymptotic synaptic weight, 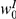, in learning dynamics of a single inhibitory synapse is shown as a function of **A** the ratio between potentiation and depression, 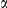 (for 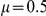) **B** the parameter 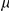 (for 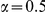). The open blue squares show the results obtained in simulating the stochastic learning dynamics using an integrate and fire postsynaptic neuron (see Methods). The solid red line shows the fixed point of equation (14), calculated using the function 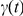 that was obtained from the fit to the spike triggered average, Figure 2. The dashed blue line shows the fixed point solution to equation (14) using a value for 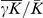 that was optimized to best fit the simulation results.

We simulated the learning dynamics of a single inhibitory synapse, keeping the rest of the inhibitory and excitatory inputs to the cell fixed. Figure 3 shows the temporal evolution of the empirical distribution of the synaptic weight. The empirical distribution was obtained by averaging over 1999 realizations of the stochastic learning dynamics of a single inhibitory synapse with uniformly distributed initial conditions. As expected from theory, the synaptic weight converges to a single fixed point, 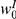, regardless of initial conditions or noise realization. Figures 4**A and B** show the dependence of the asymptotic synaptic weight, 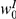, on different parameters of the learning dynamics. The solid red lines show the theoretical prediction; i.e., the fixed point of equation (14), that was calculated using the function 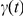 that was obtained from the fit to the spike triggered average, Figure 2. The dashed blue lines show the fixed point solution to equation (14) using an optimized value for 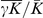 to best fit the simulation results.

### Learning a feed forward inhibitory synaptic population

Before deriving the full model for studying the learning dynamics of both feed-forward excitatory and inhibitory synapses, it is instructive to first study the artificial case of learning only the inhibitory inputs. We model a population of 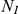 inhibitory synaptic weights, 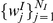, from 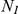 presynaptic inhibitory neurons projecting onto a single postsynaptic cell. Let us denote by 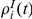 the spike train of the 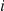
th presynaptic inhibitory neuron: 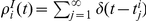 where 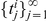 are the spike times of cell 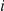 (we shall omit the subscript pre hereafter). As noted above, the cross-correlation function between the pre and postsynaptic cells is an important quantity that affects the neural dynamics. In the previous section we calculated the cross-correlation function using a linear approximation to an ‘exact’ model. Instead, here and in the following section we will use an exact solution of a simplified linear model of a more abstract neuron. For analytical tractability the postsynaptic response, 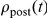, is modeled to be a delayed linear sum of its inputs: 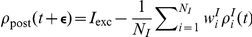; where 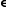 is small and positive to ensure causality, and 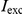 represents a constant excitatory input to the cell. For simplicity we assume that the correlations are instantaneous, 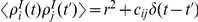. We shall further assume that the statistics of the input neuron responses are isotropic; i.e., no input neuron is statistically special. This assumption implies that: 1) The mean firing rate of all inhibitory presynaptic neurons is equal, 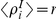. 2) The correlation structure of each input neuron with the rest of the input population (up to a permutation of indices) is the same. In particular, the correlation of a single input neuron with the total response of the population, 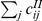, is equal for all input neurons; hence, the uniform vector 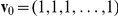 is an eigenvector of the matrix 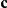. We obtain:
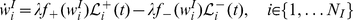
(15)

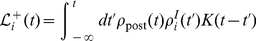
(16)

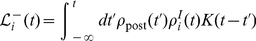
(17)As above, in the limit of slow learning rate, 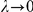, we can neglect the fluctuations of the synaptic weights around their mean, yielding
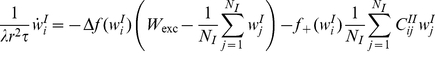
(18)where 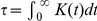, 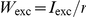, and 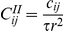 is a non-negative symmetric matrix. From the assumption of isotropy the uniform vector 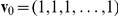 is an eigenvector of the correlation matrix, 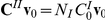, with eigenvalue 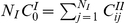.

From the assumption of isotropy, a homogeneous solution, 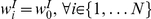, to the dynamics exists and obeys
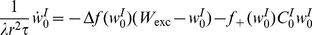
(19)with 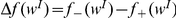. The fixed point equation for the homogeneous solution, 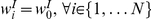, is given by
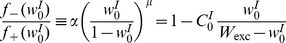
(20)The left hand side of equation (20), 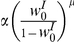, starts from 0 at 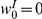, increases monotonically in the range of 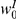, 
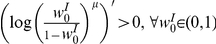
, and diverges to infinity as 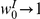. The right hand side of equation (20) decreases monotonically 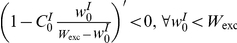, starts from the value of one at 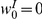, crosses zero at 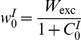 and is continuous in the range 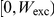. Hence, equation (20) has a unique solution, 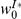, in the range of 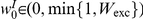 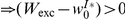, implying a net positive input to the postsynaptic cell. For 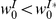 the temporal derivative of the homogeneous solution, equation (19), will be positive, and for 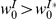 it will be negative. Hence, the uniform solution is stable to fluctuations in the uniform direction. For 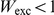, equation (20) may have an additional solution with 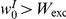. This solution is not physical, because 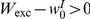 represents the case where the net input to the postsynaptic cell is inhibitory. A neuron with net inhibitory input will not fire and there will be no learning.

To study the stability of the homogeneous solution to general perturbations, we consider an arbitrary (though small) deviation from the homogeneous solution, 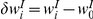 (note that we omitted the 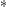 in the notation of the uniform solution). To first order in the deviations one obtains:
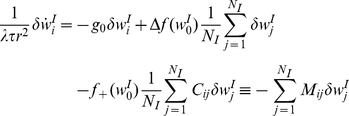
(21)

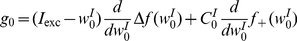
(22)

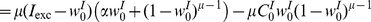
At the homogenous fixed point, equation (20), one obtains
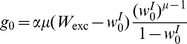
(23)Hence, 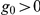. The eigenvalues 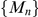 of the stability matrix 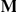 obey
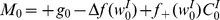
(24)

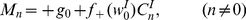
(25)where 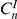 is an eigenvalue of 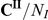, and 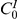 is the specific eigenvalue in the uniform direction. For stability against fluctuations in the uniform direction, see above. At orthogonal directions 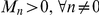 since 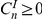 due to the positivity of the correlation matrix 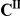. Hence, due to the negative feedback of Hebbian learning of inhibition, the uniform solution is always stable.

The STDP learning rule is an unsupervised learning rule and as such can learn salient features of the statistics of its inputs. The input statistics are expressed in the learning dynamics, equation (18), by the effective interactions between the synapses generated via the input correlations, 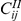, and the learning dynamics. Such sensitivity to input statistics may be manifested in solutions to the fixed point of the synaptic dynamics, equation (18), that reflect the correlations' structure of the input population. However, the homogenous solution, in which 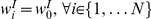, always exists from the assumption of isotropy and is stable. Thus, unlike the learning dynamics of excitatory synapses, temporally asymmetric Hebbian learning stabilizes the homogeneous solution.

#### Numerical simulations of inhibitory STDP

**Figure 5:**
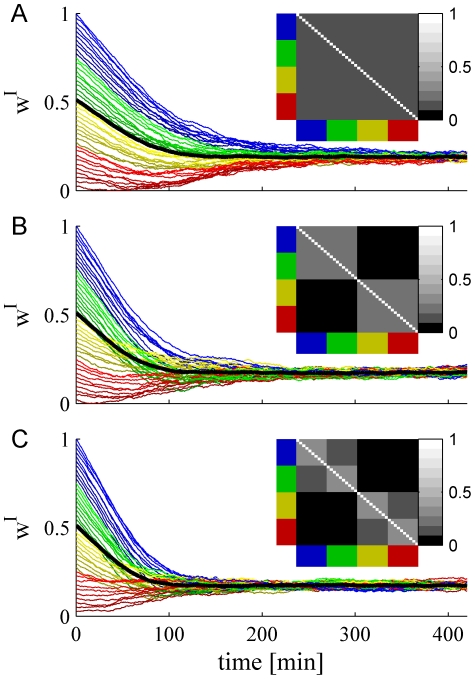
The stochastic learning dynamics of a population of 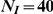 pre synaptic inhibitory neurons. Each trace shows the dynamics of a single synaptic weight. We color cells 1–10 in blue, 11–20 in green, 21–30 in yellow, and 31–40 in red. The firing rate statistics of the inhibitory neurons followed Poisson statistics with a mean rate of 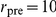 spikes/s. Initial conditions were distributed evenly from 1 to 0 for cells 1to 40, respectively, i.e., 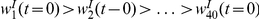. Thus, cells from the ‘blue’ population have higher initial conditions than cells from the ‘green’ population and so on. The different hues of each color distinguish the cells on the basis of their initial conditions. The thick black line shows the population average of the synaptic weights. Panels A, B and C differ in the correlation structure of the pre-synaptic neurons, shown in the inset. **A** *Homogeneous population* with uniform correlations. The correlation coefficient between all inhibitory cell pairs was 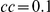 (see Methods). **B** *Heterogeneous population*. The population of 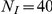 inhibitory neurons was composed of two homogenous sub-populations of 20 cells each (sub-population one: blue and green, sub-population two: yellow and red). We used a correlation coefficient of 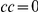 between all cells from different sub-population and uniform correlation coefficient of 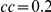 between cells from the same sub-population (see Methods). **C** *Heterogeneous population*. The population of 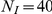 inhibitory neurons was composed of four homogenous sub-populations of 10 cells each. The different colors distinguish the cells belonging to the different populations. The correlation coefficient within each sub-populations was 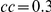. The correlation coefficient between cells in the blue sub-populations and the green sub-populations, and pairs from yellow and red was 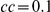. All other correlation coefficients were zero. Here we used 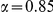, 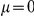, and 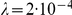.

Numerical simulations corroborate the claim that the negative feedback of inhibitory plasticity stabilizes the homogeneous solution. Figures 5**A, B and C** show two examples of the stochastic learning dynamics of a population of 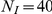 inhibitory synapses (the values of the excitatory synaptic weights were held fixed). The weight of every synapse is depicted as a function of time. In the example in Figure 5**A** the system is *homogeneous* with uniform correlation structure between all inhibitory presynaptic neurons, with a correlation coefficient of 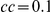 (see inset). After a transition period, which scales linearly with the learning rate, all memory of their initial conditions are lost and the system converges to a uniform solution. Figure 5**B** shows an example of a *non-homogeneous* system, where the inhibitory presynaptic population is composed of two sub-populations of equal size with a correlation coefficient of 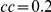 between cells from the same sub-population and a correlation coefficient of 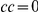 between cells from different sub-populations (see inset). The different sub-populations are depicted by different hues of red and yellow versus green and blue and are also distinguished by the range of their initial conditions. Nevertheless, as can be seen from the figure, the homogeneous solution remains stable, in line with the above analysis. Moreover, because Hebbian learning of inhibition induces negative feedback, the non-uniform correlations accelerate the convergence to a uniform solution (compare Figure 5**A and 5B**). Figure 5**C** shows another example of a *heterogenous* population with a more elaborate correlation structure (see inset), yet the homogeneous solution of the STDP dynamics remains stable.

**Figure 6:**
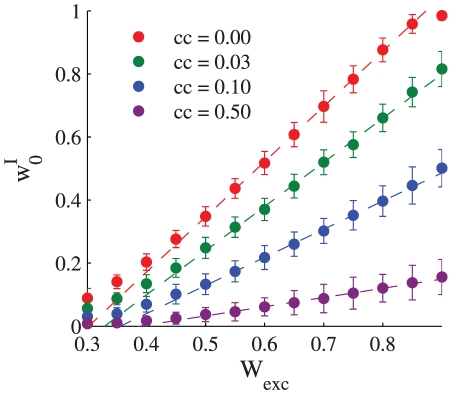
Balance of excitation and inhibition. The asymptotic value of the uniform synaptic coefficient, 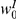 is shown as a function of the total excitatory input to the cell, for different levels of uniform correlations between the presynaptic inhibitory neuron population, 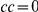, 0.03, 0.1, and 0.5 from top to bottom. The strength of the excitatory synapses were uniform and were held fixed during each simulation. In this simulation we used 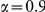, 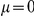, and the correlations between the inhibitory cells were uniform. The dashed lines show linear regression lines for comparison. The regression was computed using only the points with 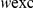 that were in the range of [0.5, 0.85].

**Figure 7:**
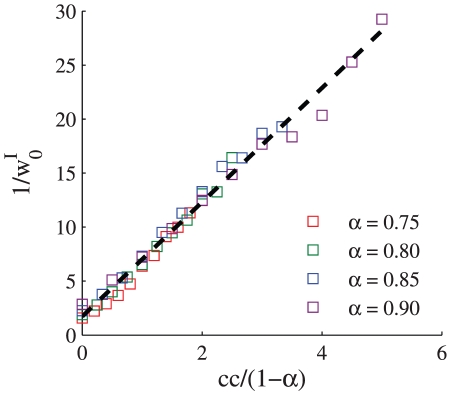
The effect of correlations on the excitation and inhibition balance. The asymptotic value of the uniform synaptic coefficient, 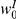 is shown as a function of the level of the homogeneous correlations in the firing of the inhibitory presynaptic population, for different values of 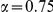, 0.8, 0.85, and 0.9 for red, green, blue, and purple, respectively. The dashed line shows the linear interpolation of the data, 
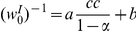
; 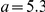 and 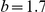. In this simulation we used 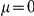, the correlations between the inhibitory cells were uniform and were varied in the range 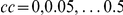, for every value of 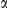.

The fixed point, equation (20), for the 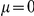, is given by
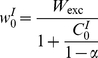
(26)
Figure 6 shows the asymptotic value of the learned synaptic uniform weights 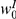 as a function of the strength of the excitatory input to the postsynaptic cell, 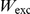. The deviations from the linear relation at low levels of excitatory input result from the non-linearity of the integrate and fire neuron used in the simulations. In particular, note that the linear interpolations (dashed lines) do not cross the abscissa at the origin, but rather at a positive value, representing the effect of a positive threshold in the 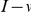 curve (i.e., input current vs. output firing rate) of the postsynaptic neuron. Figure 7 shows the asymptotic value of the learned synaptic uniform weights 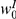 as a function of the strength of the uniform correlation coefficient between the inhibitory presynaptic cells.

### Learning feed forward inhibitory and excitatory ‘synaptic populations’

We now turn to generalize the above formalism to study the effect of Hebbian inhibitory synaptic plasticity in the framework of a simplified model for learning 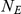 excitatory synapses, 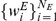, and 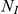 inhibitory synapses, 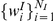, constituting feed-forward input to a single postsynaptic cell. We denote by 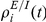 the spike train of the 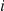
th excitatory/inhibitory neuron, 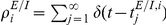 where 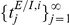 are the spike times of the cell. As above, the postsynaptic response, 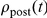, is modeled to be a delayed linear sum of its inputs: 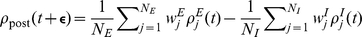 (where 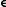 is small and positive). We further assume that the system is isotropic, the mean firing rates are equal for all presynaptic neurons, 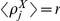 (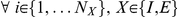), correlations are instantaneous, 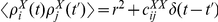, and inhibitory and excitatory inputs are uncorrelated. The excitatory synapses also follow temporally asymmetric Hebbian spike timing dependent plasticity according to equations (1)–(2) with 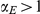 and 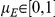. In the limit of a slow learning rate the mean field synaptic dynamics are given by
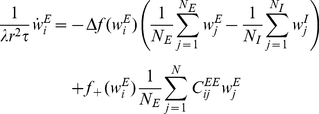
(27)

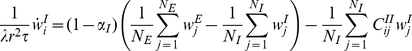
(28)where 
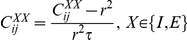
. Note that equation (28) uses 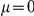 for the inhibitory synapses. The homogeneous solution, 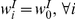 and 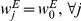, to the fixed point equations of the dynamics always exists, and obeys:
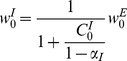
(29)

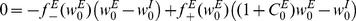
(30)where from our assumption of isotropy the uniform vector is an eigenvector of the non-negative symmetric matrix 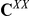 (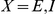) with the corresponding eigenvalue 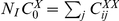. Fluctuation analysis around the homogeneous fixed point yields:
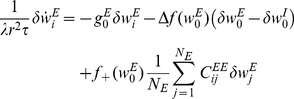
(31)

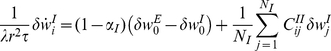
(32)

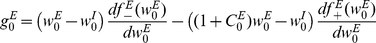
(33)

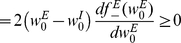
Let us denote by 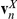 the eigenvectors of 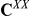 (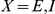) with the corresponding non negative eigenvalue 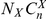; note that 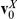 is the uniform 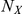 dimensional vector. The eigenvectors of the full stability matrix, 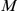, which is given by equation 
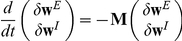
, are of the form 
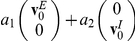
, and 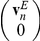, 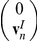 for 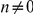.

Using the fixed point equations (29)–(30) one can show that the homogeneous solution is always stable to fluctuations in the homogeneous direction. Additionally, similar to the analysis of the previous section, the homogeneous solution is always stable to fluctuations in directions of modifying the inhibition, 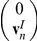. However, the homogeneous solution is not always stable with respect to fluctuations in non-homogeneous directions of the excitation. This point has been discussed at length in [28], [29], [42]. Essentially, as the positive feedback of the STDP dynamics of the excitatory synapses becomes strong (i.e., for small 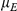) the homogeneous solution of the excitatory synapses loses its stability and the learning dynamics becomes more sensitive to the correlation structure of its excitatory inputs. Specifically, the eigenvalues of the stability matrix 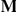 in the directions of non-homogeneous fluctuations of the excitatory synapses, 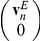 (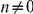), are: 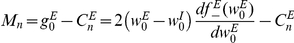. The term 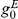 has to stabilize non-homogeneous fluctuations in the 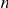
th excitatory direction, 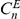. For small 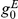 and sufficiently large 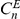 the homogenous solution will lose its stability. In addition, note that here 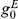 is proportional to the deviation from the balance 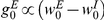, which is governed by the correlations between the inhibitory inputs 
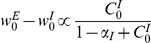
. As the correlations between the inhibitory neurons decrease the net input to the postsynaptic cell becomes more balanced and thus the homogeneous solution becomes less stable.

Nevertheless, since the learning dynamics of the inhibitory neurons, equation (28), is only sensitive to the mean *excitatory* input, 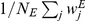, and the directions of instability of the homogeneous solution are only in a heterogenous direction of the excitatory synapses and not the inhibitory, inhibition is still expected to remain uniform, obeying:
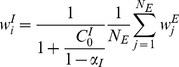
(34)Hence, importantly, we find from equation (34) that the negative feedback of inhibitory plasticity works to *balance* the net excitatory input to the cell. In the absence of cross-correlations between inhibitory synapses, 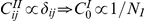, our model predicts that Hebbian STDP dynamics converge to a complete balance of excitation and inhibition 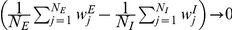, in the limit of large 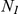. In the presence of correlations, the net inhibitory input will scale linearly with the net excitatory input (for 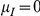); however, with a coefficient that is less than one. In this case; i.e., in the presence of correlations, the deviation from exact balance will scale with the magnitude of the fluctuations. Thus, the fluctuations in the feed forward synaptic inputs to the cell will not be negligible relative to their mean.

#### Numerical simulations of inhibitory and excitatory STDP

**Figure 8:**
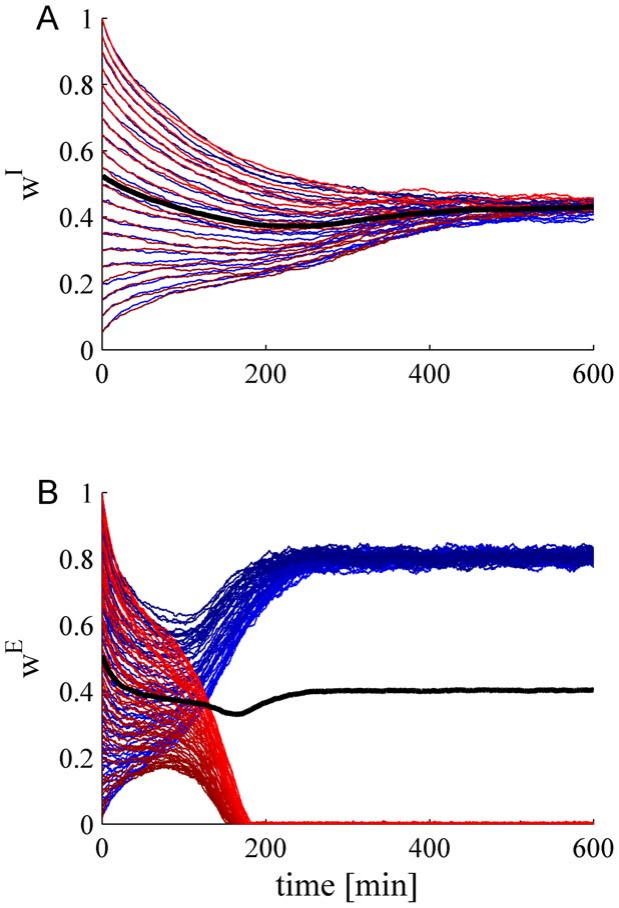
The learning dynamics of a population of 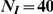 inhibitory and 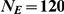 excitatory presynaptic neurons. Each trace shows the temporal evolution of a single synaptic weight for: **A** Inhibitory population **B** Excitatory population. The firing rate statistics of the presynaptic neurons followed Poisson process statistics with a mean rate of 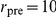 spikes/s. Each group of excitatory and inhibitory populations was composed of two sub groups of equal sizes with a uniform correlation coefficient within each group of 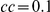 and a zero correlation coefficient between cell pairs from different sub groups (see Methods). The subgroups are distinguished by the different colors red and blue. There were no correlations between excitatory and inhibitory neurons. The thick black line shows the population average of the synaptic weights. Here we used 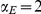, 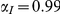, 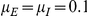, and 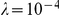.

We simulated the learning dynamics of a population of 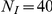 inhibitory and 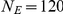 excitatory feed forward synapses onto a single integrate and fire post synaptic cell. Figure 8 shows an example in which every population (excitatory and inhibitory) is composed of two correlated subpopulations. As can be seen from the figure, although the correlation structure destabilizes the homogeneous solution to the excitatory synapses, Figure 8**B**, the homogeneous solution to the inhibitory synapses remains stable.

**Figure 9:**
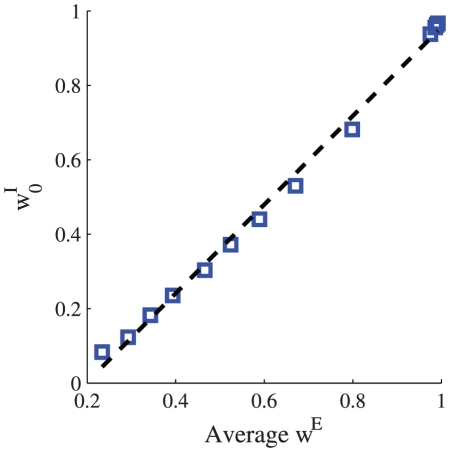
Excitation inhibition balance. The asymptotic value of the homogeneous inhibitory synaptic weight is shown as a function of the asymptotic mean excitatory synaptic weights, 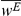. Learning dynamics of a population of 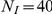 inhibitory and 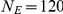 excitatory feed forward synapses onto a single integrate and fire post synaptic cell were simulated (see Methods). The firing rate statistics of the presynaptic neurons followed a Poisson process with uniform rates of 10 spikes/s. The firing of different inhibitory neurons were taken to be independent, whereas the excitatory neurons were modeled to have uniform correlations. To obtain different values for the mean excitatory input to the cell we varied the level of the uniform correlations between all presynaptic excitatory neurons 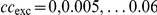, from bottom to top. Here we used the following parameters 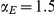, 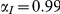, and 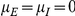. The dashed line shows a linear regression line, for comparison.

Figure 9 shows the dependence of the homogeneous inhibitory asymptotic weight on the asymptotic population average of the excitatory weights, 
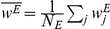
. Note that since the integrate and fire neuron is not a ‘linear’ neuron, as was used for the analysis, an exact linear relation is not expected. For example, the linear regression line (dashed line) crosses the abscissa at positive 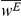, which is a manifestation of a threshold effect of the 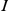-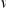 (the relation between input current and output mean rate) curve of the postsynaptic neuron. Nevertheless, the inhibitory synaptic weights are uniform and increase monotonically with the mean excitatory input.

**Figure 10:**
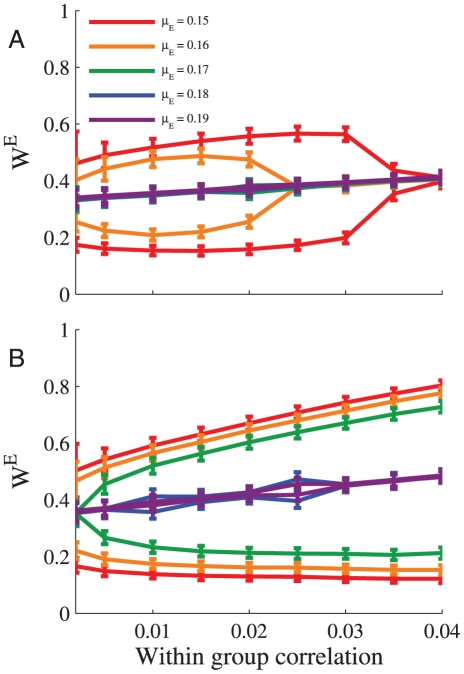
Sensitivity of excitatory plasticity. The learning dynamics of a population of 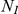 inhibitory and 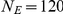 excitatory presynaptic neurons was simulated. The firing rate statistics of the presynaptic neurons followed Poisson process statistics with a mean rate of 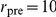 spikes/s. The inhibitory population was homogeneous and without correlations. The excitatory population was composed of two subgroups of equal size with a uniform correlation coefficient within each group, 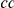, and a zero correlation coefficient between cell pairs from different subgroups (see Methods). The figure shows the mean synaptic weight of each excitatory subgroup (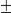 standard deviation) at the end of the learning process, as a function of the within-group correlation strength for different values of 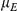. **A** Without inhibition, 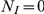. **B** With learning of a homogenous population of 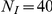 inhibitory synapses. Here we used 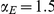, 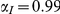, 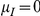, and 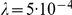. The points on the graph represents the mean over the last 600 minutes, simulation time was 2400 minutes.

The sensitivity of excitatory plasticity to the statistical structure of the presynaptic input layer is illustrated in the example of Figure 8**B**: The homogenous solution loses its stability and the synaptic weights are segregated according to the correlation structure of two competing subgroups. Figure 10 shows the difference in the mean excitatory synaptic weight of each such subgroup as a function of within-group correlation coefficient (between-group correlations were zero). The sensitivity of the learning dynamics can be thought of as the degree in which the correlation structure is express in the resultant weights. As 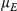 is increased the difference between the two subgroups decreases, and the sensitivity vanishes. However, the learning dynamics is more sensitive to the correlation structure of the excitation with inhibitory plasticity (Figure 10**B**) than without inhibition (Figure 10**A**). Nevertheless we note that: 1. Although the effect of increased sensitivity exists, it does not appear to be dramatic in the numerical simulations (note the values of 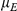). 2. The increased sensitivity results from the presence of the inhibition and not necessarily from their learning dynamics (examine the stability analysis of the homogeneous solution, above).

## Discussion

We have studied the computational effect of temporally asymmetric Hebbian plasticity of feed forward inhibition. Hebbian plasticity of inhibition generates negative feedback, in contrast to the positive feedback generated by Hebbian plasticity of excitation. This can be understood by the following intuitive explanation. If the feed forward inhibitory synapse is very strong, then it is less likely that a postsynaptic spike will follow a presynaptic spike. As a result more pre-post spike pairs will fall on the acausal branch of the STDP learning curve than on the causal branch. This, in turn, will depress the strong synapse. On the other hand, if the synapse is weak, then pre and post spike times will be largely uncorrelated and the STDP dynamics will sample uniformly both branches of the STDP curve with equal probability. If the area under the acausal branch is smaller than the area under the causal branch, 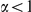 (for 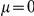), the weak synapse will potentiate (the case of 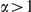 is not interesting since it reduces all inhibitory synapses to zero).

This negative feedback, in the case of temporally asymmetric Hebbian plasticity and instantaneous correlations as was studied here, implies that the inhibitory synaptic weight distribution is unimodal and that the homogeneous solution of learning a population of *inhibitory* synapses is stable, even when the homogenous solution for the excitatory synapses loses its stability. However, in our analysis we focused on a simple case where there are no correlations between excitatory and inhibitory neurons. Incorporating such correlations to our model adds a term of the form 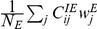 to the right hand side of equation (28). This term will cause the homogeneous solution for the inhibition to cease to exist when the uniform solution to the excitation loses its stability. Yet the increased stability of the homogeneous solution of the inhibition suggests that the inhibitory feed forward input to a cell is expected to be more broadly tuned than the excitatory input.

In addition we found that inhibitory Hebbian plasticity works to balance the net excitatory inputs of the cell. Two terms govern the homogeneous fixed point of the inhibitory synapses. The first term results from the contribution of the product of the mean firing rates to the pre-post full-correlations(this is a generalization of the fixed point equation of equation (28) to the 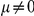 case): 
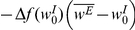
, where 
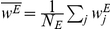
. The second term results from the contribution of covariation in the firing: 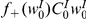. The first term, works to balance the net inhibitory and excitatory inputs to the post-synaptic cell. In the absence of correlations between the pre-synaptic inhibitory neurons, 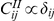, the contribution of the covariance term will decay as 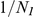. In this case, neglecting the covariance term, the inhibitory fixed point will balance the net excitatory input: 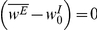, as long as the net excitatory input is not too large: 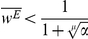. Thus, in the absence of correlations, Hebbian STDP of inhibition will balance the excitation, even for the 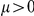 case.

In the presence of correlations, for the 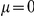 case, inhibition will balance excitation in the sense that it will scale linearly with it, this balance is skewed towards excitation, as was reported, e.g., in Heiss et al. [9]. For 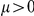, inhibition will not scale linearly with excitation. However, the deviation from exact balance (i.e., 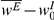) is expected to scale (not necessarily linearly) with the magnitude of the fluctuations, 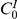, for both the 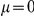 and 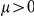 cases.

The balance has a twofold effect. First, balancing the mean excitation and inhibition inputs to the cell increases the relative contribution of the fluctuations to the cell's response. Note that we find that even if exact balance is not obtained the fluctuations are not expected to be negligible relative to the mean input. Second, the exact balance reduces the stability of the homogeneous solution to the learning dynamics to fluctuations of excitatory synaptic weights in a non-homogeneous direction. Hence, it increases the sensitivity of the STDP dynamics to the structure of the excitatory input.

Our analysis was performed using the framework of simplified models of postsynaptic neural response. Although these simplified models provide a mere caricature of the rich response dynamics of a neuron to its synaptic inputs, they further our analytical understanding of the possible outcomes of Hebbian inhibitory synaptic plasticity, which cannot be achieved if the neural response dynamics need to be solved numerically. Furthermore, numerical simulations support our analytical results qualitatively beyond the framework of the simplified linear neuron model.

We introduced several additional simplifying assumptions to our model. First, we focused on temporally asymmetric Hebbian STDP for the inhibitory synapses. However, STDP rules may be highly variable and this variability may be manifested by a qualitative difference from our results. For example an anti-Hebbian STDP rule, such as depicted in Figure 1B, may generate positive feedback to the inhibitory plasticity, instead of the negative feedback reported here. Woodin et al. [49] reported a temporally *symmetric* STDP rule in which the learning rule acts as a coincidence detector of post- and pre-spikes, Figure 1D. This learning rule may act to shape the timing of postsynaptic cell spikes by ‘selecting’ the inhibitory inputs that fired in a specific time interval around the excitatory input. Such a mechanism would require a specific temporal correlations structure between the input inhibition and excitation.

Of particular interest is the ‘thalamocortical’ circuit, in which feed forward excitatory inputs arrive directly from thalamus to cortex whereas inhibitory inputs result from a relay of the thalamic input via local (cortical) interneurons. This network architecture manifests in a rich temporal and functional (similarity of preferred stimuli) correlation structure between excitation and inhibition, which may have a significant qualitative effect on learning dynamics. However, here we assumed a very basic correlations structure between the responses of the presynaptic neurons. The investigation of the different effects of various STDP rules and the elaborate spatial and temporal correlations structure of presynaptic neuron responses is beyond the scope of the current paper. Nevertheless, this work suggests a theoretical framework for addressing these issues.

## Methods

### Details of numerical simulations

#### The leaky integrate and fire model

We simulated learning dynamics of feed forward synaptic inputs into a single post synaptic integrate and fire cell. Following Song et al [37] and Gütig et al [42] the dynamics of the membrane potential of the postsynaptic cell, 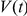, obey:
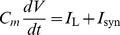
(35)

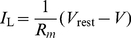
(36)

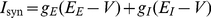
(37)where 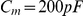 is the membrane capacitance, 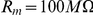 is the membrane resistance, the resting potential is 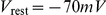, and the excitatory and inhibitory reversal potentials are 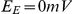 and 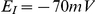, respectively. An action potential is fired once the membrane potential crosses the firing threshold of 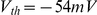, after which the membrane potential is reset to the resting potential. The synaptic conductances, 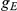 and 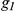 are given by
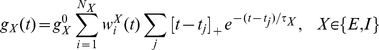
(38)where 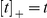 for 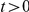 and 0 otherwise. For convenience we used 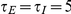 ms.

For the conductance, we introduced a scaling mechanism on the values used by Gütig et al [42] with the following rationale. The synaptic conductances, 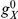, were scaled with a scaling factor, 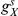, that decreased with the size of the population: 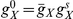. For the excitation Gütig et al [42] used 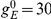 nS for 1000 excitatory synapses, and 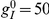 nS and 200 inhibitory synapses. We used the same 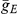 and 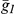, and in order to have the same average electric current in different synaptic population sizes we used 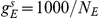 and 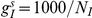 (the fact that there is 1000 in the numerator instead of 200 is explained below). To illustrate the above let us examine the case where the excitation 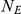 is taken to be 1000. In this case 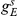 will be 1 and it will match the working point of [42]. When inhibition 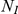 is taken to be 200, the scaling factor, 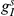 will be 5. The reasoning for this amplification is that in [42] the inhibitory synapses were held constant at their maximum value 1, whereas we are interested in a dynamic range for our inhibitory synapses to enable learning.

#### Details of numerical simulations

The synaptic spike trains to the integrate and fire neuron were simulated by Bernoulli processes (i.e., binary vectors - see below for details on generating these vectors) defined over discrete time bins of duration 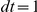 ms. These vectors are then linearly filtered using a discrete convolution kernel in the shape of 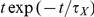 with limited length of 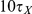 (after which this kernel function is zero for all practical purposes) to generate the right side sum of equation (38).

Integration of the synaptic and leak currents in equation (38) to estimate the postsynaptic membrane potential, 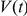, was done using the Euler method with the same step size of 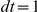 ms.

The firing rate statistics of all presynaptic cell activity throughout all our simulations followed Poisson statistics with stationary mean firing rates of 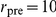 spikes/s. For the generation of instantaneously correlated Poisson point processes presynaptic activity (where applicable) we followed Gütig et al [42]. Using their defined mechanism to choose and generate the matrices for specific synaptic sub-group guarantees that within this sub-group the spike trains have the desired firing rates and instantaneous pairwise correlation coefficients.

#### The learning rate

For the learning rate, 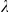, equation (1), we used two approaches as explained below. For purposes of illustration, e.g., Figures 3, 5, and 8, where we were interested in showing the learning dynamics, we used a constant learning rate throughout the entire simulation. In those cases where we were only interested in obtaining the asymptotic value to which the synaptic weights converge we accelerated the learning dynamics by using the following learning rate approach. The simulation code was flexible to support a given vector of 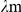 for each minute unit. Specifically we used the following formula to generate this vector.: 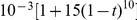, where 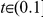, is the ratio between the minute iteration time and the entire simulation time. Examining the behavior of this function shows that it starts from a value of 16e-3 and decays significantly fast towards 1e-3, leaving the trailing 70% of the simulation with more or less the same learning rate of about 1e-3.
